# Associations of Physical Activity and Dietary Fat Quality With Arterial Health in Adolescents

**DOI:** 10.1111/ijpo.70048

**Published:** 2025-08-04

**Authors:** Mika Jormanainen, Aino‐Maija Eloranta, Marja H. Leppänen, Tomi Laitinen, Mika Kähönen, Emilia Laitinen, Timo A. Lakka, Eero A. Haapala

**Affiliations:** ^1^ Sports and Exercise Medicine, Faculty of Sport and Health Sciences, University of Jyväskylä Jyväskylä Finland; ^2^ Institute of Public Health and Clinical Nutrition, School of Medicine, University of Eastern Finland Kuopio Finland; ^3^ Institute of Biomedicine, School of Medicine, University of Eastern Finland Kuopio Finland; ^4^ Department of Medicine, Endocrinology and Clinical Nutrition Kuopio University Hospital, Wellbeing Services County of North Savo Kuopio Finland; ^5^ Faculty of Medicine, University of Helsinki Helsinki Finland; ^6^ Institute of Clinical Medicine, University of Eastern Finland Kuopio Finland; ^7^ Diagnostic Imaging Center, Kuopio University Hospital Kuopio Finland; ^8^ Department of Clinical Physiology Tampere University Hospital Tampere Finland; ^9^ Faculty of Medicine and Health Technology, Tampere University Tampere Finland; ^10^ Foundation for Research in Health Exercise and Nutrition, Kuopio Research Institute of Exercise Medicine Kuopio Finland; ^11^ Children's Health and Exercise Research Centre, Public Health and Sport Sciences, University of Exeter Medical School, University of Exeter Exeter UK

**Keywords:** arterial stiffness, exercise, youth

## Abstract

**Background:**

While the clinical signs of cardiovascular diseases (CVDs) are not usually visible until adulthood, the CVD pathology begins already in childhood.

**Objectives:**

To study the associations of physical activity (PA) and dietary fat quality with arterial health among adolescents.

**Methods:**

Altogether 117 adolescents 15–17 years of age participated in the study. Sedentary time (ST), light PA, moderate‐to‐vigorous PA (MVPA), PA energy expenditure (PAEE), resistance training volume and dietary fat quality were assessed. Pulse wave velocity (PWV) and cardio‐ankle vascular index (CAVI) were assessed by impedance cardiography, and carotid artery intima‐media thickness (cIMT) and carotid artery distensibility were assessed by carotid ultrasonography.

**Results:**

ST was negatively associated with cIMT (standardised regression coefficient *β* = −0.225, *p* = 0.015). MVPA and PAEE were negatively associated with PWV (*β* = −0.245 to −0.228, *p* < 0.05) and CAVI (*β* = −0.226 to −0.212, *p* < 0.05), and positively with cIMT (*β* = 0.235 to 0.269, *p* < 0.05). MVPA was positively associated with carotid artery distensibility (*β* = 0.180, *p* = 0.047). Monounsaturated fat intake was positively associated with carotid artery distensibility (*β* = 0.190, *p* = 0.041). PAEE was negatively associated with CAVI in adolescents with higher saturated fat (SFA) intake (*β* = −0.367, *p* = 0.017), but not in adolescents with lower SFA intake (*β* = −0.095, *p* = 0.526).

**Conclusion:**

MVPA and PAEE were related to better arterial health. Moreover, our results suggest that higher PAEE benefits adolescents with higher SFA intake.

## 1 Introduction

Cardiovascular diseases (CVDs) are the most common non‐communicable diseases worldwide [1, 2, 3, 4]. CVDs typically become apparent in middle adulthood [1]. However, pathophysiological processes causing atherosclerosis and early arterial stiffening can start already in childhood [1, 5]. The first signs of atherosclerosis and arterial stiffening, such as increased carotid intima‐media thickness (cIMT) and pulse wave velocity (PWV), have been found to independently predict clinical cardiovascular events, stroke and cardiovascular mortality [6].

Higher levels of physical activity (PA) and better diet quality, particularly the quality of dietary fat, have been considered key modifiable factors in reducing CVD risk [7]. Alarmingly, only 19% of adolescents 11–17 years of age achieve the recommended levels of PA [8] and 34% exceed the recommended levels of daily saturated fat (SFA) intake in Nordic countries [9]. However, there is limited evidence on the independent and combined associations of dietary fat quality and PA with arterial health in adolescence.

Higher PA levels have been associated with lower arterial stiffness and less advanced subclinical atherosclerosis in youth [10]. However, most studies have considered only endurance type PA and assessed PA using questionnaires [11]. Accordingly, resistance training has been related to lower levels of adiposity and other cardiometabolic risk factors [12, 13, 14], but there are no previous studies on the associations of resistance training with arterial health among adolescents. Moreover, PA intensity may be an important consideration in the associations between PA and arterial health, as moderate to vigorous PA has been found to have stronger associations with arterial health than light PA in youth [15, 16, 17]. However, a few studies have investigated the relationships between PA intensity and valid measures of arterial health in adolescents [15].

In adults, a higher SFA intake has been associated with advanced arterial stiffening and atherosclerotic progression [18]. However, only energy percentage from dietary fats, not SFA, was directly associated with arterial stiffness in children aged 10 years [19]. Nevertheless, the evidence on the associations between dietary fat quality and arterial health, primarily measured by PWV and augmentation index (AIx) in the paediatric populations, is based on a very small number of studies and therefore, the associations of dietary fat quality with arterial health should be interpreted cautiously [3]. Although dietary fat quality may also modify the associations between PA and arterial health [3], to the best of our knowledge, there are no previous studies investigating the role of dietary fat intake in the associations between PA and arterial health in adolescents.

The independent associations of PA and dietary fat quality with arterial health as well as the modifying effect of dietary fat quality on the associations between PA and arterial health in adolescents remain poorly understood. Therefore, we investigated the independent associations of PA and dietary fat quality with arterial health and studied the modifying effect of dietary fat on the associations between PA and arterial health.

## 2 Materials and Methods

### 2.1 Study Design and Participants

The present cross‐sectional data are from the 8‐year follow‐up of the Physical Activity and Nutrition in Children (PANIC) Study, which is a PA and dietary intervention and follow‐up study in a population sample of children from the city of Kuopio, Finland [20]. The arterial health measurements were performed only at the 8‐year follow‐up and therefore we analysed the cross‐sectional associations of PA and diet with arterial health outcomes. Altogether 736 children 6–8 years of age from primary schools of Kuopio were invited to participate in the baseline examination in 2007–2009. A total of 512 children, representing 70% of those invited, participated in the baseline examinations. Six children were excluded from the study at baseline because of physical disabilities that could hamper participation in the intervention or no time or motivation to attend the study. We also excluded data from two children whose parents or caregivers later withdrew their permission to use these data in the study [21]. The participants did not differ in sex distribution, age, or body mass index standard deviation score (BMI‐SDS) from all children who started the first grade in 2007–2009 based on data from the standard school health examinations performed for all Finnish children before the first grade (data not shown). Subsequently, 504 children were invited to a 2‐year follow‐up study between 2009 and 2011, of whom 438 (87%) participated [22]. Finally, 277 children participated in the 8‐year follow‐up in 2016 and 2017.

The Research Ethics Committee of the Hospital District of Northern Savo approved the study protocol in 2006 (Statement 69/2006) and amended it in 2015 (Statement 422/2015). The parents or caregivers of the children gave their written informed consent, and the children provided their assent to participation. The PANIC study has been carried out in accordance with the principles of the Declaration of Helsinki as revised in 2008.

### 2.2 Assessment of PWV and CAVI

PWV and CAVI were measured with the CircMon B202 impedance cardiography device (JR Medical Ltd., Saku Vald, 419 Estonia). The participants were asked to rest for 15 min in a supine position before the measurement. Next, current electrodes were placed on the distal parts of the extremities, slightly proximal to the wrists and the ankles. Voltage electrodes were placed proximal to the current electrodes, with a distance of 5 cm between the centers of the electrodes. The distal impedance plethysmography was recorded from a popliteal artery at the knee joint level. The active electrode was placed on the lateral side of the knee joint, and the reference electrode on the calf. The distance between the electrodes was about 20 cm. The distance between the electrode sites was measured using unstretchable measurement tape. Alternating electrical current was applied to current electrodes and the change in whole‐body impedance was measured from voltage electrodes. The CircMon B202 software estimates the foot of the impedance cardiography signal that coincides with pulse transmission in the aortic arch and the foot of the impedance plethysmography signal that coincides with pulse transmission in the popliteal artery [23].

### 2.3 Assessment of cIMT and Carotid Arterial Distensibility

For the assessment of cIMT and distensibility of the left common carotid artery, carotid ultrasound imaging was performed utilising the Acuson Sequoia 512 Ultrasound Mainframe (Acuson, Mountain View, CA, USA) with a 14.0 MHz linear array transducer using a standardised protocol. The sonographers analysed the ultrasound scans offline from the digitally stored images. From the image, at least three measurements of the far wall of the common carotid artery were taken approximately 10 mm proximal to the bifurcation to determine the maximal carotid IMT [24]. For the assessments of carotid artery distensibility, the diameter of the common carotid artery at end‐diastole and end‐systole was measured at least twice. In addition, the sonographer measured systolic blood pressure (SBP) and diastolic blood pressure (DBP) from the brachial artery just before and directly after the ultrasound scans. The means of the end‐diastolic and end‐systolic diameters, as well as SBP and DBP values, were used to calculate arterial distensibility indices. Carotid arterial distensibility was calculated as: ((systolic − diastolic diameter)/diastolic diameter)/(SBP‐DBP) [20].

### 2.4 Assessment of Physical Activity and Sedentary Time

A uniaxial accelerometer with a built‐in heart rate sensor (Actiheart, CamNtech Ltd., Papworth, UK) was attached to the chest via ECG electrodes and used to assess sedentary time (ST) and PA. The device was set to record body movement and heart rate in 60‐s epochs [25, 26]. The participants were instructed to carry on with their usual behaviour and to wear the monitor during all daily activities, including sleep, shower, sauna, and swimming [25]. The participants were requested to wear the device continuously for a minimum of four consecutive days: two on weekdays and two on the weekends [27]. We accepted ST and PA data for statistical analyses if there were at least 48 h of activity recording in weekday and weekend day hours that included at least 12 h from morning (3 AM–9 AM), noon (9 AM–3 PM), afternoon (3 PM–9 PM), and night (9 PM–3 AM) to avoid potential bias from over‐representing specific times and activities of the days [25]. Upon retrieving and downloading the data from the device, heart rate data were first corrected for noise. The time of waking up was defined as accelerometer counts increasing and remaining above zero and heart rate increasing and remaining above the plateau level. Subsequently, they were individually calibrated with sleeping heart rate and parameters obtained from maximal exercise tests performed by the Ergoselect 200K electromagnetic bicycle ergometer (Ergoline, Bitz, Germany) and the Cardiosoft V6.5 Diagnostic System ECG device (GE Healthcare Medical Systems, Freiburg, Germany) [25]. The heart rate data were finally combined with trunk acceleration data in a branched equation model to estimate activity intensity time series [28]. Monitor non‐wear was acknowledged by prolonged zero acceleration lasting > 90 min accompanied by non‐physiological heart rate, and activity estimates were adjusted during summarisation to minimise diurnal bias arising from non‐wear. Physical activity energy expenditure (PAEE) was calculated by integrating the intensity time series, where time distribution of activity intensity was generated by using standard metabolic equivalents (METs) in 0.5 increments. Sleep duration was analysed from the Actiheart recordings by a trained exercise specialist and confirmed by a physician, where necessary [25]. The time of falling asleep was defined as accelerometer counts decreasing to zero and heart rate to a plateau level. The time of waking up was defined as accelerometer counts increasing and remaining above zero and heart rate increasing and remaining above the plateau level.

We defined ST as time spent in activity ≤ 1.5 METs excluding sleep and light PA, moderate PA and vigorous PA as time spent in activity > 1.5 and ≤ 4.0 METs, > 4.0 and ≤ 7.0 METs and > 7.0 METs, respectively, by defining 1 MET as 71.2 J/min/kg. MVPA included moderate PA and vigorous PA [25].

Time spent in resistance training was assessed using the PANIC Physical Activity Questionnaire for Adolescents. The adolescents were asked to report how often they had participated in resistance training during the past year. The participants were asked to report the weekly frequency and duration of resistance training (min/day) in sports clubs, other supervised practises, or unsupervised training. These were summed up to represent the general resistance training volume and used in the analyses. We also divided the adolescents into those participating in resistance training at least three times per week and those participating in resistance training less than three times per week based on the current PA guidelines [29].

### 2.5 Assessment of Dietary Fatty Acids

We assessed the consumption of food and drinks and the intake of nutrients using food records [30]. The food records covered four predefined and consecutive days, including two weekdays and two weekend days or three weekdays and one weekend day. The clinical nutritionists gave instructions about the food records to the study participants at the research site during the study visits. A clinical nutritionist checked the returned food records together with the adolescents and filled in any missing information. We calculated food consumption and nutrient intake using the Micro Nutrica dietary analysis software, version 2.5. The software is based on detailed information about the nutrient content of foods in Finland and other countries [31]. Moreover, a clinical nutritionist updated the software by adding new food items and products with their actual nutrient content based on new data in the Finnish Food Composition Database [32] or received from the producers [31].

### 2.6 Assessment of Body Size and Body Composition, and Somatic Maturity

Whole‐body mass was measured twice, with the children having fasted for 12 h, emptied the bladder, and standing in light underwear by a calibrated InBody 720 bioelectrical impedance device (Biospace, Seoul, South Korea) to an accuracy of 0.1 kg. The mean of these values was used in the analyses. Stature was measured three times with the children standing in the Frankfurt plane, without shoes, using a wall‐mounted stadiometer to an accuracy of 0.1 cm. The mean of the nearest two values was used in the analyses. BMI was calculated by dividing body mass (in kilogrammes) by body height (in square metres). BMI–SDS was calculated based on Finnish reference data [33]. Total fat mass and body fat percentage (BF%) were measured by the Lunar dual‐energy x‐ray absorptiometry device (GE Medical Systems, Madison, WI) using standardised protocols [34]. Somatic maturity status in terms of time to peak height velocity was calculated using the equations by Moore et al. [35].

### 2.7 Statistical Analysis

All statistical analyses were performed with IBM SPSS 28 statistics (IBM Corp. NY, Armonk, USA) software. The characteristics of participants were presented as means (standard deviations) or medians (interquartile range) for continuous variables with skewed distribution. The associations of measures of ST, LPA, MVPA, VPA, PAEE, resistance training and dietary intake of SFA, monounsaturated fat (MUFA) and polyunsaturated fat (PUFA) with arterial health were investigated using linear regression analyses adjusted for age, sex and somatic maturity. The data were further adjusted for BF%, energy intake and SBP and DBP. To study the modifying effect of dietary fat quality on the associations of PA with arterial health was investigated including the dietary fat × PA interaction term was included in the models. The data from the linear regression analyses are presented as unstandardized regression coefficients (B), their 95% confidence intervals (CIs), and standardised regression coefficients (*β*). *p*‐values < 0.05 were considered statistically significant. Differences in PWV, CAVI, cIMT and carotid artery distensibility between adolescents participating in resistance training at least three times per week and those participating in resistance training less than three times per week were analysed using general linear models. The data were corrected for multiple comparisons using the Benjamini Hochberg false discovery rate (FDR). We considered FDR corrected *p*‐values ≤ 0.2 significant, as a more stringent threshold would impair the balance between type I and type II errors [36].

## 3 Results

### 3.1 Participants

Altogether 117 adolescents 15–17 years of age (48 girls and 69 boys) had valid data on all required variables and were included in the final analyses. Participants included in the analyses did not differ in age, sex distribution, somatic maturity, BMI‐SDS, or BF% at 8‐year follow‐up from those excluded from the analyses (all *p* > 0.068). Only one participant reported participation in resistance training at least three times per week. Therefore, comparisons between those participating in resistance training at least three times per week and those participating in resistance training less than three times per week were not performed. The basic characteristics of the participants are presented in Table 1.

**TABLE 1:** Characteristics of study participants.

	All (*n* = 117)	Girls (*n* = 48)	Boys (*n* = 69)
Age (years)	15.8 (15.0 to 17.4)	15.8 (15.0 to 16.4)	15.8 (15.1–17.4)
Height (cm)	171.6 (147.9 to 194.7)	166.6 (147.9 to 179.3)	176.5 (161.6–194.7)
Weight (kg)	61.7 (41.2 to 124.4)	56.6 (41.2 to 78.3)	66.7 (43.0–124.4)
BMI (kg/m^2^)	20.9 (15.7 to 38.7)	20.4 (16.1 to 27.4)	21.3 (15.7–38.7)
BMI‐SDS (mean, min–max)	−0.07 (−2.39 to 2.80)	−0.13 (−2.06 to 1.69)	−0.01 (−2.39 to 2.80)
Time to relative PHV (years)	2.75 (0.80 to 4.41)	3.41 (2.42 to 4.41)	2.08 (0.80–3.45)
ST (min/day)	603 (223 to 918)	608 (306 to 838)	597 (223–918)
LPA (min/day)	324 (113 to 691)	319 (105 to 563)	329 (113–691)
MVPA (min/day)	47 (2 to 165)	39 (2 to 143)	54 (3–165)
VPA (min/day)	11 (0 to 78)	7 (0 to 51)	15 (0–78)
PAEE (kJ/kg/day)	52.3 (16.4 to 141.7)	47.2 (17.5 to 100.3)	57.3 (16.4–141.7)
RT in a sports club (min/day)	0.10 (0.00 to 1.70)	0.00 (0.00)	0.20 (0.00–1.70)
RT other training (min/day)	0.15 (0.00 to 15.80)	0.00 (0.00)	0.30 (0.00–15.80)
RT unsupervised training (min/day)	8.2 (0.0 to 61.6)	7.9 (0.0 to 53.3)	8.4 (0.0–61.6)
RT total (min/day)	8.3 (0.0 to 78.9)	7.9 (0.0 to 53.3)	8.7 (0.0–61.6)
Energy intake (Kcal/day)	1870 (811 to 4065)	1658 (908 to 3448)	2081 (811–4065)
SFA intake (g/day)	25.8 (9.9 to 56.5)	22.6 (9.2 to 56.1)	29.0 (9.9–56.5)
MUFA intake (g/day)	24.5 (8.4 to 60.1)	22.0 (8.4 to 46.0)	27.0 (8.9–60.1)
PUFA intake (g/day)	13.2 (3.5 to 29.8)	12.4 (5.0 to 29.8)	13.9 (3.5–29.5)
PWV (m/s)	5.8 (5.1 to 8.5)	5.8 (5.1 to 8.1)	5.8 (5.1–8.5)
CAVI	6.4 (4.7 to 13.5)	6.5 (4.7 to 13.5)	6.2 (4.1–12.8)
cIMT (mm)	0.44 (0.29 to 0.64)	0.43 (0.29 to 0.50)	0.45 (0.31–0.64)
Carotid artery distensibility (%/10 mmHg)	2.75 (1.33 to 5.31)	2.91 (1.90 to 5.31)	2.59 (1.33–4.99)

Abbreviations: BMI, body mass index; BMI‐SDS, body mass index standard deviation score; CAVI, cardio‐ankle vascular index; cIMT, carotid intima‐media thickness; LPA, light physical activity; MUFA, monounsaturated fat; MVPA, moderate to vigorous physical activity; PAEE, physical activity of energy expenditure; PUFA, polyunsaturated fat; PWV, pulse wave velocity; RT, resistance training; SFA, saturated fat; ST, sedentary time; VPA, vigorous physical activity.

### 3.2 Independent Associations of Physical Activity and Dietary Fat With Arterial Health

MVPA and PAEE were negatively associated with PWV and CAVI (Table 2). MVPA, VPA and PAEE were positively associated with cIMT. In contrast, ST was negatively associated with cIMT. MVPA and MUFA intake were positively associated with carotid artery distensibility. These associations remained statistically significant after further adjustment for BF%, energy intake and SBP and DBP. Resistance training was not associated with arterial health (Table 2). These associations remained after FDR correction.

**TABLE 2:** Association of physical activity and dietary fat with PWV, CAVI, cIMT and carotid artery distensibility.

	PWV (m/s)				CAVI				cIMT (mm)				Carotid artery distensibility (10^−3^ × mmHg^−1^)			
	*B*	95% CI	*p*	*β*	*B*	95% CI	*p*	*β*	*B*	95% CI	*p*	*β*	*B*	95% CI	*p*	*β*
ST (min/day)	0.001	0.000 to 0.002	0.088	0.175	0.002	0.000 to 0.004	0.126	0.161	**0.000**	0.000 to 0.000*	**0.015**	**−0.225**	0.000	−0.001 to 0.001	0.478	−0.064
LPA (min/day)	−0.001	−0.002 to 0.000	0.072	−0.185	−0.002	−0.005 to 0.001	0.126	−0.162	7.987E‐5	0.000 to 0.000	0.135	0.140	0.000	−0.001 to 0.001	0.818	0.021
MVPA (min/day)	**−0.003**	−0.007 to 0.001*	**0.028**	**−0.228**	**−0.008**	−0.017 to 0.000*	**0.047**	**−0.212**	**0.000**	0.000 to 0.001*	**0.012**	**0.235**	**0.004**	0.000 to 0.007*	**0.047**	**0.180**
VPA (min/day)	−0.007	−0.015 to 0.001	0.084	−0.184	−0.021	−0.042 to 0.001	0.059	−0.206	**0.001**	0.000–0.002*	**0.032**	**0.205**	0.008	−0.001 to 0.017	0.088	0.158
PAEE (kJ/kg/day)	**−0.006**	−0.010 to −0.001*	**0.018**	**−0.245**	**−0.014**	−0.026 to −0.001*	**0.034**	**−0.226**	**0.001**	0.000 to 0.001**	**0.004**	**0.269**	0.004	−0.002 to 0.009	0.182	0.122
RT total (min/day)	0.007	−0.001 to 0.015	0,071	0.184	0.017	−0.005 to 0.038	0.136	0.156	−0.001	−0.002 to 0.000	0.059	−0.176	−0.008	−0.017 to 0.001	0.082	−0.157
SFA (g/day)	−0.007	−0.020 to 0.006	0.310	−0.115	−0.019	−0.055 to 0.017	0.295	−0.122	0.000	−0.002 to 0.001	0.721	−0.036	0.010	−0.004 to 0.024	0.162	0.134
MUFA (g/day)	−0.009	−0.022 to 0.004	0.192	−0.142	−0.021	−0.057 to 0.015	0.247	−0.129	0.001	−0.001 to 0.002	0.293	0.102	**0.015**	0.001 to 0.030*	**0.041**	**0.190**
PUFA (g/day)	−0.018	−0.039 to 0.003	0.097	−0.173	−0.036	−0.094 to 0.023	0.227	−0.130	0.000	−0.002 to 0.002	0.860	0.017	0.020	−0.005 to 0.044	0.112	0.144

*Note: p*‐value under the limit of *p < 0.05 and **p < 0.01. Statistical significant association are bolded.

Abbreviations: LPA, light physical activity; MUFA, monounsaturated fatty acids; MVPA, moderate‐vigorous physical activity; PAEE, Physical Activity Energy Expenditure; PUFA, polyunsaturated fatty acids; RT total, resistance training total; SFA, saturated fatty acids; ST, sedentary time; VPA, vigorous physical activity.

### 3.3 Modifying Effect of Dietary Fat on the Associations of Physical Activity With Arterial Health

PAEE was negatively associated with CAVI in adolescents with higher SFA intake (*B* = −0.020, 95% CI = −0.036 to −0.004, *β* = −0.367 *p* = 0.017 for interaction), but not in adolescents with lower SFA intake (*B* = −0.007, 95% CI = −0.029 to 0.015, *β* = −0.095). The positive association between PAEE and cIMT was stronger in adolescents with lower PUFA intake (*B* = 0.001, 95% CI = 0.000 to 0.002, *β* = 0.327, *p* = 0.019 for interaction) than among those with higher PUFA intake (*B* = 0.001, 95% CI = 0.000 to 0.001, *β* = 0.243). Further adjustment for BF% attenuated the association between PAEE and CAVI in adolescents with higher SFA intake (*B* = −0.014, 95% CI = −0.027 to −0.001, *β* = −0.237). Additionally, further adjustment for energy intake with SFA, MUFA and PUFA did not affect the results (*p* > 0.05). These associations remained after FDR correction.

## 4 Discussion

We observed that MVPA and PAEE were negatively associated with PWV and CAVI and that MVPA and MUFA intake were positively associated with carotid artery distensibility. VPA was not associated with PWV or CAVI. Conversely, higher levels of MVPA and VPA and lower levels of ST were associated with higher cIMT. However, resistance training was not associated with arterial health. Interestingly, PAEE was negatively associated with CAVI in adolescents with a higher intake of SFA, but such association was not observed among those with lower SFA intake. Our findings indicate that engaging in at least moderate intensity PA may have a beneficial association with arterial stiffness and that this association may have a stronger association with arterial stiffness among adolescents with unhealthier dietary fat composition.

In line with previous studies [10, 11, 37], we found that MVPA was more strongly associated with lower arterial stiffness than LPA, suggesting that habitual PA should be intense enough to improve arterial health [10]. However, VPA was not associated with arterial stiffness. The reason for this weak association may be that adolescents accumulated, on average, only 11 min of VPA per day. The underlying reason behind the inverse relationship of MVPA to arterial stiffness is likely complex and is associated with various beneficial effects of increased PA, such as reduced fat mass, oxidative stress and systemic inflammation [38] and increased nitric oxide production and upregulation of endothelial nitric oxide synthase activity [39]. Moreover, PA can reduce systemic low‐grade inflammation in children with poorer diet quality [40]. Arterial stiffness develops with fractures in elastic tissue due to repeated cyclic stress [41, 42]. This process is complicated by systemic inflammation and free radicals, which lead to media layer hyperplasia and calcification of the smooth muscle layer, resulting in impaired endothelial function [41, 42].

cIMT has been found to predict clinical cardiac events and stroke [5] and exercise training has been found to decrease cIMT in adults. We found, counterintuitively, that MVPA and VPA were positively associated with cIMT. Some evidence suggests that long‐term, high‐volume and high‐intensity exercise can accelerate atherosclerosis progression. Therefore, our findings may reflect the influence of long‐term MVPA and VPA behaviour on blood flow patterns, proinflammatory response, or calcium homeostasis that are associated with atherosclerosis progression. However, this is merely speculative, as direct evidence in children and adolescents is not available. On the other hand, the association of MVPA and VPA with cIMT may also reflect adaptive remodelling in response to haemodynamic load rather than pathological atherosclerotic changes [43]. Moreover, Agbaje found that fat‐free mass mediated the direct association between MVPA and cIMT. Fat‐free mass has been positively associated with both carotid lumen diameter and cIMT, resulting in the preservation of the cIMT/lumen ratio and arterial circumferential wall stress [44]. Thus, increased fat‐free mass in response to PA may also partially explain our findings. However, more studies investigating whether MVPA‐ and VPA‐induced changes in cIMT influence long‐term cardiovascular risk across the life course are warranted.

In line with a previous systematic review [45], we observed weak, if any, associations between ST and arterial stiffness. However, we found that ST was inversely associated with cIMT, disagreeing with the results of the previous review showing no association between ST and cIMT. However, aligning with the results of our study, Agbaje found an inverse association between ST and cIMT in 1574 adolescents [46]. The mechanisms explaining these results can only be speculated, but reduced blood flow and shear stress leading to lower haemodynamic load [47] and thereby less arterial remodelling may be one reason why sedentary adolescents had lower cIMT than their less sedentary peers. Altogether, these results may indicate that increased levels of ST may not be a main driver of atherosclerotic processes in adolescence. However, it is important to consider that atherosclerotic changes occur earlier in the abdominal aorta than in the carotid arteries, and therefore cIMT may not be a sensitive measure to detect early atherosclerotic changes [48].

We found that a positive association exists between MUFA intake and carotid artery distensibility, indicating a higher elasticity of the carotid artery. An improved ratio of saturated to unsaturated fat has been favourably associated with aortic distensibility in adolescents aged 11–19 years [49]. Cumulative exposure to dietary SFA from childhood to adulthood has been directly associated with aortic IMT but not cIMT [49]. However, we found that SFA was not independently associated with arterial stiffness or cIMT. Nevertheless, SFA intake modified the association between PA and arterial health. PAEE was negatively related to arterial stiffness assessed by CAVI among adolescents with higher SFA intake. However, PAEE had weak, if any, associations with CAVI among those with a lower SFA intake. These results imply that SFA may negatively impact arterial health, particularly in adolescents with lower PA levels. Whereas the exact mechanisms for our observation remain unknown, SFA can expand white adipose tissue and increase oxidative stress and inflammation already in adolescence [16], but higher levels of PA may mitigate these harmful effects.

Whereas resistance training has been found to decrease body fat content and improve other cardiometabolic risk factors, such as insulin sensitivity, little is known about its association with arterial health [12, 13, 14]. We observed that resistance training was not associated with arterial health in adolescents, suggesting that it may not induce major vascular adaptations in youth. Our findings align with previous observations that aerobic exercise and high‐intensity interval training elicit larger beneficial effects on arterial stiffness than resistance training in children and adolescents [50]. Therefore, these findings together suggest that endurance‐type PA rather than resistance training should be promoted to improve arterial health in youth. However, the research evidence is still limited, and further studies are warranted.

The strengths of our study include valid and reproducible methods to assess PA, ST and dietary fat quality. There are also some limitations that need to be noted when interpreting the results. Firstly, the sample size in the final statistical analyses was rather small. Secondly, cross‐sectional analyses do not provide evidence about causality. Thirdly, PA was assessed by the Actiheart monitor with 60‐s epochs, which may underestimate the time spent in PA and ST. Moreover, Actiheart is placed on the chest as opposed to being on the thigh, the latter of which would provide a more accurate assessment of ST [25, 26]. Moreover, we assessed resistance training using a questionnaire, which did not capture information on training intensity. Fourthly, 4‐day food records are considered the gold standard for assessing habitual dietary intake. The 4‐day food records were carefully checked and completed by trained clinical nutritionists to minimise possible misreporting. However, subjective reporting is always prone to bias. Participants may have altered their diet intentionally or unintentionally during the recording process. Moreover, participants may have underreported particularly foods that are considered ‘unhealthy,’ such as fatty foods. This may have affected the estimated fat intake [51]. Finally, our participants were from a general population of Caucasian adolescents, so our results may not be directly generalisable to other populations.

## 5 Conclusion

We observed that total PA and particularly MVPA were positively associated with arterial elasticity in adolescents. Additionally, our findings suggest that higher levels of PAEE benefit arterial elasticity, especially in adolescents with higher SFA intake. Moreover, resistance training was not related to arterial health. Therefore, our findings highlight incorporating moderate‐intensity PA in habitual PA to promote arterial health in adolescents.

## Conflicts of Interest

The authors declare no conflicts of interest.

## Data Availability

The data that support the findings of this study are available on request from the corresponding author. The data are not publicly available due to privacy or ethical restrictions.
